# A Fabricated Foot Fracture Caused by Deliberate Placement of an External Osseous Fragment: A Case Report

**DOI:** 10.7759/cureus.117100

**Published:** 2026-09-28

**Authors:** Laith Hseinat, Majdala Al-Bataineh, Hamza A Abu-Ain, Refat AlRawashdeh, Qais Sharayah, Fayiz Samarah, Mutaz Jaloody, Qusai Alqarqaz, Ashraf Albadaineh, Mahmoud Sbaihat

**Affiliations:** 1 Orthopaedics, Jordanian Royal Medical Services, Amman, JOR; 2 Faculty of Medicine, Yarmouk University, Irbid, JOR; 3 Orthopaedic Surgery, Jordanian Royal Medical Services, Amman, JOR; 4 Orthopaedics and Traumatology, Ministry of Health, Amman, JOR; 5 Orthopaedics and Traumatology, Jordanian Royal Medical Services, Irbid, JOR

**Keywords:** clinical–radiological correlation, diagnostic pitfall, fabricated injury, foot fracture, malingering, radiographic artifact, secondary gain

## Abstract

Accurate diagnosis of musculoskeletal injuries requires correlation between clinical and radiological findings. Although plain radiographs are essential for fracture assessment, external objects may mimic osseous pathology and lead to misdiagnosis. We present an unusual case of a fabricated foot fracture caused by deliberate placement of an external osseous fragment for secondary gain. A 23-year-old previously healthy man presented with left foot pain and inability to bear weight following a reported low-energy fall at his military base. Initial radiographs demonstrated an apparent osseous fragment adjacent to the first metatarsal, provisionally interpreted as a fracture, and the patient was treated with below-knee cast immobilization. On follow-up, repeat radiographs showed an unexpected change in the fragment’s position. Computed tomography demonstrated that the fragment was external to the foot and embedded within the cast material, with no true fracture. During supervised repeat radiography, the patient was observed deliberately positioning the fragment adjacent to the foot and subsequently admitted to fabricating the injury to obtain sick leave. Discrepancies between clinical findings and serial radiographs should prompt careful reassessment and consideration of external artifacts. Recognition of unexpected changes in the position of an apparent fracture fragment may prevent unnecessary immobilization, investigations, and treatment, particularly when secondary gain is suspected.

## Introduction

Plain radiographs remain a foundational and commonly used imaging modality for detecting or excluding fractures following trauma. Despite their established role, interpretation of skeletal radiographs can be challenging, particularly in the trauma setting. Lower-limb trauma radiographs had an overall sensitivity of 93.5% and specificity of 89.7% for initial interpretation, with a false-positive rate of 10.3%, while pooled sensitivity and specificity for foot radiographs were 94.9% and 94.0%, respectively, indicating that interpretation errors may occur despite generally high diagnostic accuracy [1,2]. The interpretation of foot radiographs can be particularly challenging because of the complex bony anatomy of the foot and overlap of multiple bones on conventional projections. Foot fractures are among the commonly missed fractures in the lower extremity, and small avulsion fractures may present additional diagnostic difficulties. Familiarity with normal anatomical appearances and careful attention to the clinical presentation are therefore important when evaluating suspected foot fractures. A systematic approach includes assessment of alignment, bones, and joint congruity, with fractures assessed for cortical discontinuity and accessory bones considered according to their location, shape, and border characteristics [2,3]. Accessory ossicles are developmental variants that may appear as small, well-corticated supernumerary osseous structures and can occur near bones or articulations. Although generally incidental, they may become clinically relevant following trauma and can resemble fracture fragments, particularly avulsion fractures, creating a potential source of diagnostic error [4-6]. Several accessory ossicles of the foot have been described as fracture mimics, including os vesalianum pedis and os intermetatarseum. Os calcaneus secundarius is another uncommon example that may resemble an avulsion fracture of the anterior process of the calcaneus on plain radiographs. In a Jordanian radiographic survey, it was identified in 0.3% of foot and ankle radiographs and was noted as a potential diagnostic mimic [4,5,7,8]. Distinguishing an accessory ossicle from an acute fracture may be challenging when relying on plain radiographs and physical examination alone. Accurate interpretation requires correlation of the radiographic abnormality with the clinical findings, particularly the location of pain and tenderness. When findings remain uncertain, CT or magnetic resonance imaging may provide additional information to establish the diagnosis. CT may be particularly useful in demonstrating the characteristic well-corticated margins and anatomical relationships of accessory ossicles and has been reported to be more sensitive than plain radiography for their detection [2,5,6,8]. Importantly, not every apparent osseous fragment represents an anatomical variant or a true fracture. When a radiographic abnormality does not correspond with the clinical examination or expected anatomy, reassessment of the finding and consideration of an external artifact may be warranted. Our case presents an unusual instance of a fabricated foot fracture in a young adult, in which an external osseous fragment was deliberately positioned adjacent to the foot and misinterpreted as a fracture fragment on initial radiographs. The case illustrates the importance of systematic clinical-radiological correlation and stepwise reassessment when an apparent fracture finding is inconsistent with the clinical and anatomical context.

## Case presentation

A 23-year-old previously healthy man presented to the emergency department in July 2026 with left foot pain following direct trauma to the foot after a low-energy fall at his military base. He reported an inability to bear weight on the affected limb. On initial assessment, the patient’s military socks were not removed because of crowding in the emergency department at that time. The examination was limited to the exposed dorsal aspect of the foot, with no plantar palpation performed. No gross deformity was noted. The patient reported ecchymosis, although this could not be directly assessed by the examining resident because the socks remained in place. Localized tenderness was reported over the dorsal first and second metatarsal regions, the first dorsal web space, and the base of the great toe. No blood was observed on the socks. Foot and ankle movements were limited by pain, while distal neurovascular findings were normal.

Plain radiographs of the left foot, including anteroposterior, oblique, and lateral views, demonstrated an apparent osseous fragment between the first and second metatarsals (Figure 1). The first through fifth metatarsals appeared intact. The radiopaque structure was initially interpreted as a displaced osseous fragment resembling a metatarsal, and an extra-metatarsal fracture was provisionally suspected. The patient was managed conservatively with below-knee cast immobilization, granted one week of sick leave, and referred to the closest fracture clinic for follow-up. There was no reported history of previous orthopaedic procedures, bone defects, or bone grafting.

**Figure 1:**
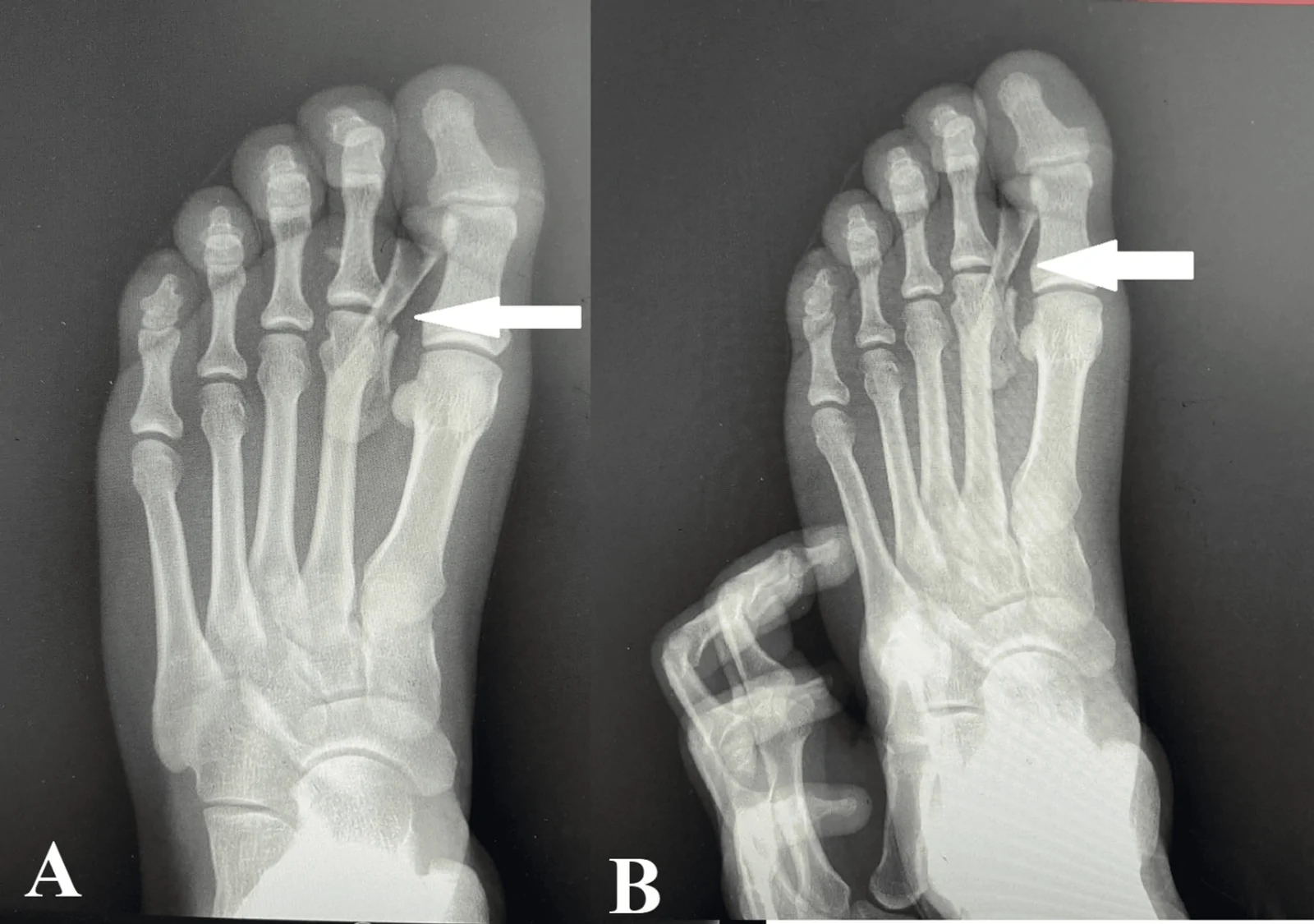
Initial Radiographic Assessment of the Left Foot Showing an Apparent Osseous Fragment Between the First and Second Metatarsals Initial radiographic assessment of the left foot. Anteroposterior (A) and oblique (B) radiographs showing a apparent osseous fragment (arrow) between the first and second metatarsal bones, with maintained alignment of the first through fifth metatarsals and preserved phalangeal alignment.

At the subsequent clinic visit, the patient continued to report severe pain and inability to bear weight and presented in a wheelchair. Repeat radiographs obtained while the below-knee cast remained in place demonstrated a noticeable change in the position of the previously identified apparent osseous fragment compared with the initial images (Figure 2). The patient continued to report pain, while distal toe capillary refill remained intact and no clinical signs suggestive of compartment syndrome were identified.

**Figure 2:**
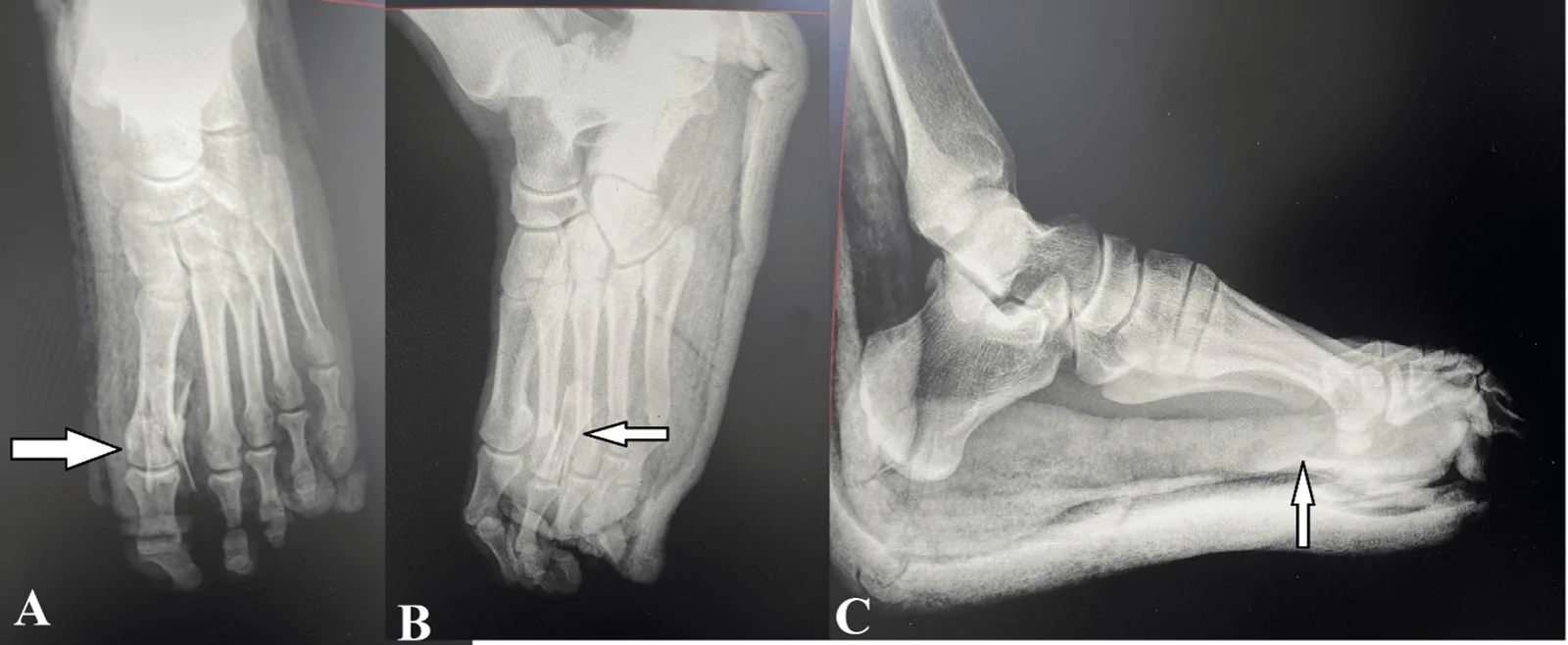
Follow-Up Radiographic Assessment of the Left Foot Demonstrating Interval Displacement of the Bone Fragment Follow-up radiographic assessment of the left foot. Anteroposterior (A), oblique (B), and lateral (C) radiographs obtained with the foot in a backslab demonstrate interval displacement of the apparent osseous fragment (arrow) toward the first metatarsal compared with the initial radiographs, while the alignment of the first through fifth metatarsals remains preserved.

Given the discrepancy between the reported symptoms and the clinical and radiographic findings, computed tomography (CT) of the left foot was requested to further clarify the diagnosis. Review of the CT images demonstrated that the radiopaque osseous fragment was located external to the foot and embedded within the cast material, with no associated fracture of the foot (Figures 3-4).

**Figure 3:**
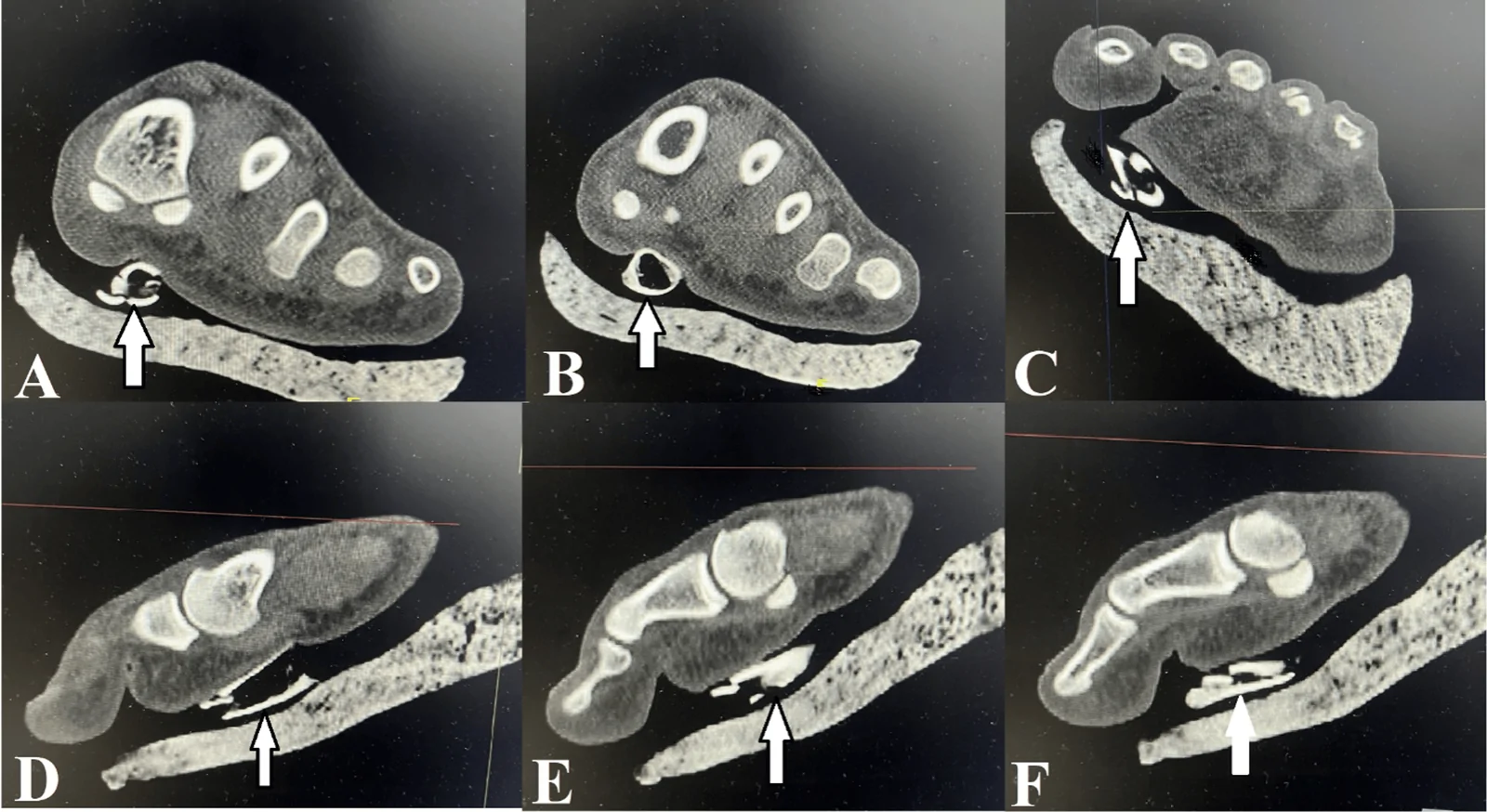
Follow-Up Computed Tomography Assessment of the Left Foot Demonstrating the External Osseous Fragment Follow-up computed tomography assessment of the left foot. Axial (A–C) and sagittal (D–F) CT images obtained with the foot immobilized in a backslab demonstrate the external osseous fragment (arrow), located outside the foot and embedded within the cast material.

**Figure 4:**
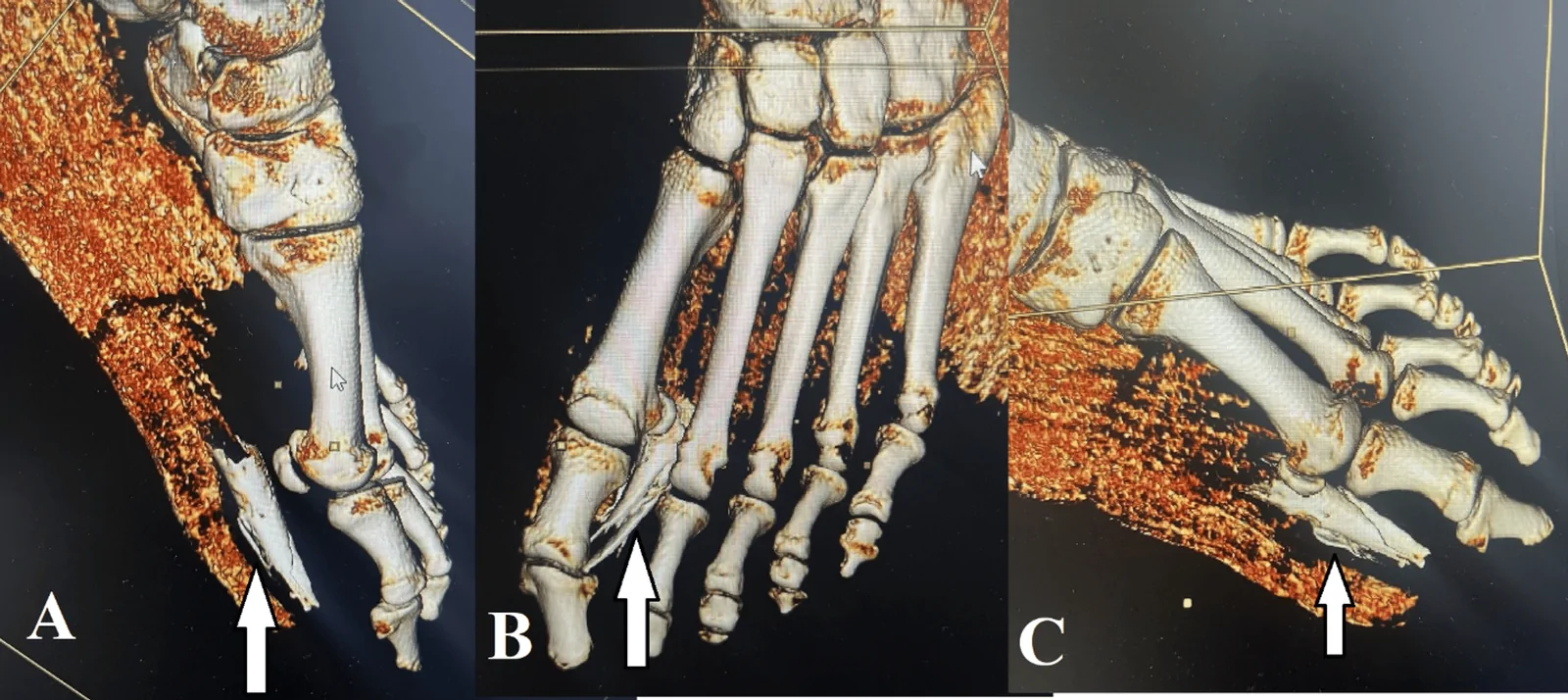
Three-Dimensional Computed Tomography Reconstruction of the Left Foot Demonstrating the External Osseous Fragment Follow-up three-dimensional (3D) computed tomography reconstruction of the left foot. Three-dimensional reconstructed CT images (A-C), obtained with the foot immobilized in a backslab, demonstrate the external osseous fragment (arrow), located outside the foot and embedded within the cast material.

The case was subsequently reviewed with senior orthopaedic specialists, and the patient was asked to return for further assessment. Supervised repeat bilateral foot radiographs were obtained (Figure 5). During imaging, the patient was observed intentionally positioning an external osseous fragment adjacent to the foot. The patient subsequently admitted to deliberately placing the fragment during the previous imaging examinations. This admission and the circumstances of the imaging were documented in the medical record.

**Figure 5:**
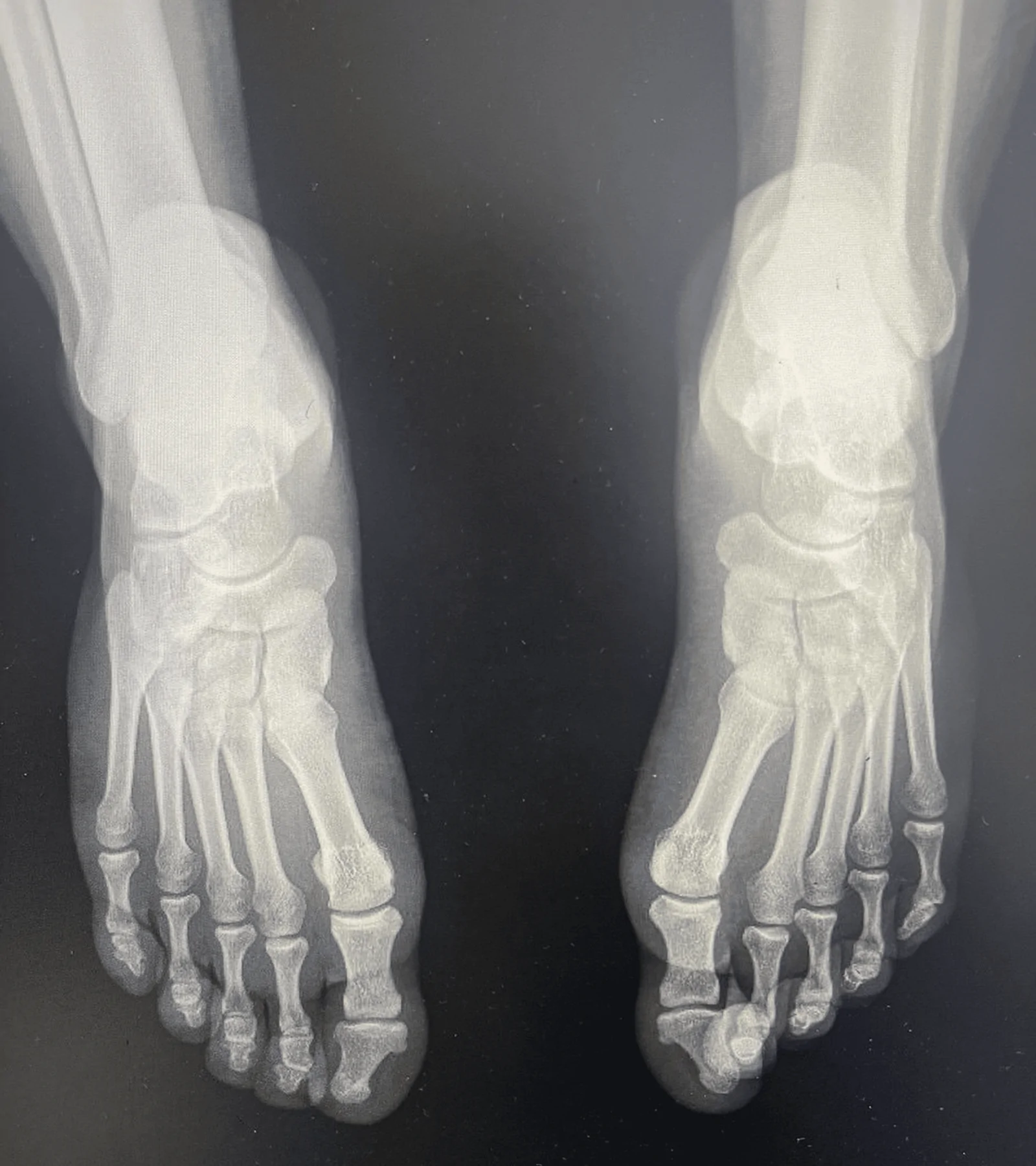
Follow-Up Bilateral Foot Radiographs After Cast Removal Follow-up bilateral foot radiographs after cast removal. Anteroposterior radiographs of both feet obtained after removal of the cast and socks demonstrate no obvious bony abnormality. The previously identified fragment is absent from its expected anatomical location, with preserved alignment of the first through fifth metatarsals, supporting that the fragment did not represent a normal anatomical structure.

No true fracture or structural injury was ultimately identified. This case highlights the importance of careful correlation between clinical examination and radiographic findings, particularly when imaging abnormalities are inconsistent with the physical findings. Unexpected changes in the position of an apparent osseous fragment on serial imaging should prompt reassessment and consideration of artifacts or external objects. Thorough physical examination, including removal of clothing, footwear, and other potential sources of external artifacts when appropriate, may prevent unnecessary immobilization, investigations, and treatment.

## Discussion

Diagnostic errors in the interpretation of trauma radiographs are multifactorial and may result from errors in perception, recognition, or decision-making. Although radiography remains the initial imaging modality for detecting or excluding fractures, interpretation may be affected by technical, anatomical, and human factors. This is particularly relevant in lower-extremity trauma involving the foot, where complex bony anatomy and overlapping structures on conventional projections can make interpretation challenging. Correlation of radiographic findings with the available clinical information and comparison with previous examinations are important components of accurate interpretation. When conventional radiographs do not adequately explain the clinical or radiological findings, additional imaging may be required to clarify the diagnosis [2,9].

In our case, the initial radiographs demonstrated an apparent osseous fragment adjacent to the first metatarsal, which was initially interpreted as a displaced fracture fragment and treated with below-knee cast immobilization. At that stage, the finding was considered in the context of the initial clinical assessment. However, the absence of significant objective findings and the subsequent change in the fragment’s position on repeat radiographs raised concern regarding the initial diagnosis. The discrepancy between the reported symptoms and the objective clinical and radiographic findings represented an important opportunity for diagnostic reassessment.

Accessory ossicles are important differential considerations when an apparent osseous fragment is identified on foot radiographs, as they may mimic fractures following trauma. Their well-corticated margins generally favor an accessory ossicle, whereas fracture margins are usually poorly corticated. Comparison with the contralateral foot and follow-up radiographs may further assist in uncertain cases. Os calcaneus secundarius, for example, can mimic a fracture of the anterosuperior calcaneal process [10]. However, our case differed from these recognized anatomical fracture mimics, as the apparent osseous fragment was not an accessory ossicle belonging to the foot.

Foreign bodies can also produce unexpected radiographic findings that may initially be attributed to other pathological conditions. A reported case of a foreign body in the foot demonstrated a misleading finding on plain radiographs that required additional imaging to clarify the diagnosis. Therefore, an external or foreign-body-related cause should be considered when an imaging abnormality does not correspond with the expected clinical or anatomical findings [2,11].

A particularly important diagnostic clue in our case was the change in the position of the apparent osseous fragment on serial radiographs. This unexpected change was difficult to reconcile with the initial interpretation of a fixed fracture fragment, particularly in the absence of corresponding clinical findings, and prompted further assessment. Comparison with previous imaging is an important component of reducing diagnostic errors, particularly when findings are unexpected or equivocal. Follow-up imaging may provide additional information that is not apparent on the initial examination and may prompt reconsideration of the original interpretation [9,10]. In retrospect, a stepwise reassessment consisting of complete exposure of the foot, repeat physical examination, and repeat radiography without external materials might have resolved the discrepancy before CT. This highlights the importance of reassessing unexpected imaging findings before proceeding to further treatment or advanced investigation.

When conventional radiographs are equivocal or do not adequately characterize an abnormality, CT can serve as a useful problem-solving modality. It can provide additional anatomical information and help distinguish fractures from their mimics or other unexpected findings. In the context of accessory ossicles, CT can also demonstrate their characteristic well-corticated appearance and anatomical relationship to adjacent structures [10].

In our case, CT provided the decisive clarification by demonstrating that the radiopaque structure was external to the foot and embedded within the cast material, with no true fracture identified. Deliberate fabrication was subsequently established when the patient was directly observed positioning an external osseous fragment adjacent to the foot and admitted to deliberately placing it during the previous radiographic examinations. This provided direct evidence that the apparent fracture was artificially created rather than a true structural injury.

Malingering and factitious disorder may both involve the intentional production or exaggeration of symptoms, but they differ primarily in motivation. Factitious disorder is associated with internal psychological gain, whereas malingering involves an external incentive. However, clinical inconsistencies alone should not automatically be considered evidence of deliberate deception, as symptom exaggeration may occur in other conditions and conscious versus unconscious exaggeration can be difficult to determine clinically. Therefore, suspected deception requires careful assessment of the clinical findings and available objective evidence [12,13]. Suspicion of secondary gain should therefore prompt careful verification rather than premature attribution of symptoms to malingering. In particular, clinical inconsistency should not lead to under-investigation of genuine pathology, as premature attribution may contribute to confirmation bias. The same principle may be relevant in other settings in which secondary gain is considered, including workers’ compensation, disability assessment, insurance claims, and medicolegal evaluations.

Overall, our case demonstrates an unusual, fabricated foot fracture in which an external osseous fragment was deliberately positioned to simulate a fracture on radiographs. The changing position of the fragment, its external location on CT, and the subsequent direct observation and admission ultimately established that no true fracture was present. The case emphasizes that unexpected discordance between the history, physical examination, and imaging findings should prompt systematic clinical and radiological reassessment before attributing the findings to a specific diagnosis or motive.

## Conclusions

This case presents an unusual example of a fabricated foot fracture caused by deliberate placement of an external osseous fragment. The discrepancy between the clinical findings and serial radiographs, together with the changing position of the apparent osseous fragment, prompted further investigation. CT demonstrated that the fragment was external to the foot, with no true fracture identified. The case emphasizes the importance of systematic clinical-radiological correlation and reassessment when history, physical examination, and imaging findings are discordant. An interval change in the position of an apparent fracture fragment should prompt consideration of a broad differential diagnosis, including technical artifact, external objects, anatomical variants, and other causes, rather than being interpreted as evidence of deliberate fabrication without objective confirmation. This principle is relevant beyond military medicine, including insurance, compensation, disability, and medicolegal settings.
